# Explaining socio-economic differences in intention to smoke among primary school children

**DOI:** 10.1186/1471-2458-14-191

**Published:** 2014-02-21

**Authors:** Henricus-Paul Cremers, Anke Oenema, Liesbeth Mercken, Math Candel, Hein de Vries

**Affiliations:** 1 Department of Health Promotion, School for Public Health and Primary Care (CAPHRI), Maastricht University, P.O. Box 616, 6200 MD Maastricht, the Netherlands; 2 Department of Methodology and Statistics, School for Public Health and Primary Care (CAPHRI), Maastricht University, P.O. Box 616, 6200 MD Maastricht, the Netherlands

**Keywords:** Socio-economic status, Intention to smoke, Primary school, Mediation analyses

## Abstract

**Background:**

Smoking prevalence is higher among low socio-economic status (LSES) groups, and this difference may originate from a higher intention to smoke in childhood. This study aims to identify factors that explain differences in intention to smoke between children living in high socio-economic status (HSES) and LSES neighbourhoods.

**Methods:**

Cross-sectional data were derived from the baseline assessment of a smoking prevention intervention study. Dutch primary school children, aged 10 – 11 years (*N* = 2,612), completed a web-based questionnaire about their attitude, subjective norm, self-efficacy expectations, modelling and intention to smoke. Linear and logistic regression analyses were performed to assess potential individual cognitive (attitude, subjective norm and self-efficacy) and social environmental (modelling) mediators between SES and intention to smoke.

**Results:**

Multiple mediation models indicated that modelling mediated the association between SES (*B* = -0.09 (*p* < 0.01)) and intention to smoke (*B* = 1.06 (*p* < 0.01)). Mainly the father, mother and other family members mediated this association. Gender did not moderate the association between SES and intention to smoke and the potential mediators indicating that there are no differences in mediating factors between boys and girls.

**Conclusions:**

This study indicates that future smoking prevention studies may focus on the social environment to prevent smoking onset. However, replication of this study is warranted.

**Trial registration:**

This study was approved by the Medical Ethics Committee of the Atrium-Orbis-Zuyd Hospital (NL32093.096.11 / MEC 11-T-25) and registered in the Dutch Trial Register (NTR3116).

## Background

Smoking is more prevalent in low socio-economic status (LSES) than in high socio-economic status (HSES) adolescents (28% vs. 18%) [1] and this difference in smoking is an important contributor to socio-economic differences in health [2]. The higher smoking prevalence among LSES adolescents and adults starts with a higher smoking initiation in childhood and early adolescence [3-7]. Since the smoking behaviour of children and young adolescents is low (0% smokes daily at age 12 in The Netherlands) [1], smoking preferences can be indicated by their intention to engage in smoking [8,9]. Currently, there is little knowledge about socio-economic status (SES) differences in smoking intention among children and factors that may explain these differences. Insight into such factors increases the knowledge base on when and where SES differences in smoking start and may provide useful target points for interventions to ultimately reduce socio-economic inequalities in smoking behaviour.

Prior research [10-12] has indicated that LSES adolescents engage more often in smoking as compared to HSES adolescents and it is likely that this difference originates from a higher intention to start smoking at a younger age. However, it is unclear which factors predict or explain the difference in intention to start smoking between LSES and HSES children. A first category of determinants that may explain these differences are individual cognitions such as attitude, subjective norm or self-efficacy expectations, because individual cognitions are involved in explaining people’s intentions (including smoking intentions) [10,12]. Even though the attitude of children toward smoking (initiation) is generally negative [13], de Vries [10] investigated a sample of Dutch students (12 – 16 years) and reported that LSES youngsters had more positive thoughts about smoking compared to HSES youngsters. HSES adolescents linked smoking more clearly with the discovery of taste and with relief from boredom while LSES adolescents perceived smoking as a way of meeting people [10]. As regards subjective norms and self-efficacy expectations an association with SES differences is less clear. Although, subjective norms might be influential since children are known to follow norms or opinions of their parents. This is not only the case with their smoking behaviour [14,15] but also regarding their intention to smoke in the future [16]. Moreover, higher self-efficacy levels have a more preventive effect for smoking initiation than lower self-efficacy levels in young adolescents [10,17,18].

Besides individual cognitions, previous studies suggested that social environmental factors might be influential in the relation between SES and the initiation of smoking among children [15,19-21]. Mainly the behaviour of important people in the direct social environment (i.e. modelling) is crucial for the formation of children’s opinions and behaviours [19]. In particular the smoking behaviour of parents, siblings, family or peers have found to be associated with the decision to start smoking among children [15,22]. Since smoking is more prevalent in LSES environments [1,10], LSES children are more often exposed to others smoking behaviour than HSES children. There is some evidence for parental and peer smoking to mediate the association between SES and the smoking behaviour of adolescents [23,24]. However, until now there is no evidence for potential mediators between SES and the intention to start smoking in children.

The aims of the present study are to examine the association between SES (measured at a neighbourhood level) and the intention to engage in smoking and to identify which factors mediate the association between SES and intention to start smoking among children aged 10 – 11 years and whether gender is a potential moderator of these associations. In this study individual cognitive (attitude, subjective norm and self-efficacy expectations) and social environmental variables (modelling) are the potential mediators of interest.

## Methods

### Sampling and procedure

Data for this cross-sectional study were gathered from the baseline measurement of a smoking prevention intervention study, called ‘Fun without Smokes’ [25], in October-November 2011. Participants in this study were children in grade 7 (10 – 11 years) of primary schools in The Netherlands. The ‘Fun without Smokes’ programme is a web-based computer-tailored smoking prevention programme that will be evaluated in a cluster randomised controlled trial (c-RCT). The baseline questionnaire was a web-based questionnaire about children’s attitude, social influences, self-efficacy expectations and intention concerning smoking. After completion of the questionnaire children in the intervention group received computer-tailored advice based on the answers provided in the questionnaire.

Children were recruited through primary schools. The primary schools were recruited by seven Dutch Municipal Health Promotion Organisations and Maastricht University. Approximately 3,500 Dutch primary schools were approached for participation and 162 schools eventually took part in the intervention study. A passive informed consent procedure was used in which children in grade 7 of all participating schools were invited to participate in the intervention trial. All parents or guardians received an information brochure of the ‘Fun without Smokes’ study. Children or their parents or guardians could indicate that they did not want to participate, by sending a resignation form to the research team (1.7% refused). In the present study children who had filled out their intention whether or not to start smoking and who had reported a complete and verifiable postal code (to calculate their SES) were included in the analyses.

The ‘Fun without Smokes’ study was approved by the Medical Ethics Committee of the Atrium-Orbis-Zuyd Hospital (NL32093.096.11/MEC 11-T-25) and registered in the Dutch Trial Register (NTR3116).

### Measurement

The primary outcome measure was *intention to smoke*. Intention to smoke was assessed by self-reports using a previously accepted staging question [26,27]. Children could indicate which one of seven statements would describe their intentions best. The statements were: ‘I am sure I will never start smoking’, ‘I think I will never start smoking’, ‘I think I will start smoking in the future’, ‘I think I will start smoking within 5 years’, ‘I think I will start smoking within 1 year’, ‘I think I will start smoking within 6 months’ and ‘I think I will start smoking within 1 month’. Children who indicated that they intended to start smoking anytime in the future were categorised as having the intention to smoke (1). Children who indicated they would never start smoking in the future were categorised as not having the intention to smoke (0).

The *SES* of the neighbourhood was based on the postal code of the participating children, which they had provided in the questionnaire. The Netherlands Institute for Social Research (Dutch government agency which conducts research into the social aspects of all areas of government policy) has gathered information on all Dutch inhabitants concerning their income, occupation and education. Those indicators were used to calculate an index score for the 4-digit postal code area. For that reason, index scores indicated the social advantage at a neighbourhood level [28,29]. A LSES environment was indicated with a low score (<0 or equal to 0) and children living in a HSES environment with a high score (>0). Index scores are well-known SES indicators for both adults and children in The Netherlands. In the present study children from a LSES environment were coded with a ‘0’ and children from a HSES environment were coded with a ‘1’. Validation analyses have shown that SES of a neighbourhood approximates well with the individual SES of Dutch inhabitants [30].

*Background variables* measured religion (1 = Catholic; 2 = Protestant; 3 = Islamic; 4 = other religion; 5 = non religion), family composition (1 = mother and father; 2 = alternately mother and father; 3 = only my father; 4 = only my mother), pocket money (yes = 1; no = 2) and school performance (0 = less well than most classmates; 1 = same as most classmates; 2 = better than most classmates) of the participating children. Additional variables measured were the age (in years), gender (1 = boy; 2 = girl) and ethnicity of the participants. A child was considered to have a Western ethnic background (1) if he/she and both parents had been born in The Netherlands, another European country, North America, Oceania, Indonesia or Japan. Otherwise the child was considered to have a non-Western ethnic background (2) [31].

#### Potential individual cognitive mediators

In line with the I-Change model [32], attitude was operationalised as consisting of both advantages and disadvantages and was separately measured in the present study. *Attitude (advantages)* measured the positive consequences of smoking with nine items. Participants answered these questions in terms of various benefits they perceived concerning smoking, such as feeling mature, sociable, cool or receiving more attention from friends. Children were able to complete the following question ‘If I smoke….’ with a four-point answer category ranging (for example) from ‘I will feel very mature (4) – I will not feel mature (1)’ (Cronbach’s alpha = 0.85).

*Attitude (disadvantages)* measured ten different negative consequences of smoking which children may perceive (such as I get less physical fit, I will become ill or I will get addicted). Children had to give an answer on a four-point scale ranging (for example) from ‘I will become very ill (4) – I will not become ill (1)’ (Cronbach’s alpha = 0.80).

*Subjective norm* measured the perception of smoking norms of important people in the child’s environment. Children had to complete seven questions including the father, mother, brother(s), sister(s), friends, best friend and most people that were important to them. An example of a question is: ‘My mother thinks that I….’. These questions had to be scored on a five-point Likert scale ranging from ‘definitely should not smoke (+2)’ to ‘definitely should smoke (-2)’ (Cronbach’s alpha = 0.69).

*Self-efficacy* expectations were measured with ten questions, assessing the ability of the child to refuse cigarettes in different situations. An example of a question which had to be completed on a five-point Likert scale ranging from ‘very easy (+2)’ to ‘very difficult (-2)’ is: ‘When others smoke it is….for me not to smoke’ (Cronbach’s alpha = 0.94).

#### Potential social environmental mediator

*Modelling* assessed the smoking behaviour of parents, siblings, family and friends. A total of eight questions were measured, such as: ‘Does your mother/father/brother(s)/sister(s)/best friend smoke?’ (five-point answering formats ranging from ‘often (5)’ to ‘never (1)’ scored these questions) and ‘How many of your friends/family members/classmates smoke?’ (five-point answering scales ranging from ‘(almost) all (5)’ to ‘(almost) none (1)’ scored these questions). Children were, furthermore, able to indicate if they had no parents, siblings, family or friends or if they did not know if people in their social environment smoked and these answers were also categorised as ‘1’. To create one variable for modelling all individual items (best friend, mother, father, brother(s), sister(s), friends, family and classmates) were summed up and divided by the number of questions to calculate an average score (ranging from 1.00 to 5.00). Therefore, a high score on this scale indicated that more people smoked in the social environment of the child.

Questions and answering formats for both potential individual cognitive mediators and the potential social environmental mediator were based on an assessment instrument that was used in a prior smoking prevention intervention trial [26,27].

### Analyses

The influence of school and class level on the smoking intentions of the participating children was analysed to test for possible nesting effects. The variance of the random intercept at school level was zero, and the variance of the random intercept at class level was not significant (*Z* = 1.56 (*p* > 0.05)), consequently, multilevel analyses were not warranted. Descriptive analyses were carried out to describe the sample under study. A correlation matrix was produced to identify correlations in potential mediators, the control variables (age, gender and ethnicity), SES and intention to smoke using the Spearman rank correlation test (*r*). Subsequently, linear and logistic regression analyses were performed to analyse whether the potential mediators (attitude, subjective norm, self-efficacy and modelling) mediated the association between SES and intention to smoke. For this analysis the joint significance test, described by Mackinnon [33] was used. Multiple mediation models, including all potential mediators, adjusted for the control variables, were used in the analysis. Pathway *a* represents the association between the independent factor (i.e. SES) and the potential mediator (individual cognitive or social environmental). In the second step the association of the potential mediator and the dependent variable (i.e. intention to start smoking) was analysed (pathway *b*). The joint significance test implies that if both pathway *a* and *b* are significant, mediation is present [34]. However, the relation between the independent factor and the dependent factor were also analysed (pathway *c*) and eventually adjusted for the potential mediators (pathway *c’*) to assess the relation between SES and intention to smoke. Differences between pathway *c* and pathway *c’* was used to determine whether there was full or partial mediation [34]. If mediation was demonstrated, multiple mediation analyses were performed to investigate which component of the individual cognitive or environmental variables explained the mediation most. All analyses were performed in SPSS 19.0 and results were considered to be significant if the *p-*value was equal to or lower than 0.05.

## Results

### Basic characteristics

A total of 2,612 children (81.3%) met the inclusion criteria and were included in the analysis. The majority of the children were of Western ethnic background (87.1%) and 46.6% were boys. Most participants received pocket money and 83.8% lived in a two-parent household. Table 1 shows that significantly more boys (62.4%) had the intention to smoke (*p* < 0.01). Children with the intention to smoke were significantly more often (*p* = 0.01) living in a LSES environment (61.3%) and had a significantly lower school performance (*p* < 0.01) than children who did not have the intention to smoke. Additionally, the minority of the pupils who had the intention to smoke had a Catholic religion (16.1%) and their family composition was significantly more often only a father or a mother (*p* < 0.01), compared to children who did not have the intention to smoke.

**Table 1:** Basic characteristics

	**Total sample (N= 2,612)**	**Intention to smoke (N= 93)**	**No intention to smoke (N= 2,519)**	***t***	***X***^**2**^	**df**	***p*-value**
Age (in years)	10.35	10.40	10.35	-0.88	-	2,543	0.38
Gender (% boy)	46.6	62.4	46.0	-	9.64	1	**<0.01**
Ethnicity (% Western)	87.1	88.2	87.0	-	0.11	1	0.75
SES (% HSES environment)	51.3	38.7	51.7	-	6.08	1	**0.01**
Pocket money (% yes)	77.5	75.3	77.6	-	0.28	1	0.60
School performance (% high)	28.8	19.4	29.2	-	15.49	2	**<0.01**
Religion (% Catholic)	20.1	16.1	20.2	-	1.66	4	0.80
Family composition (% both parents)	83.8	72.0	84.3	-	9.91	1	**<0.01**

### Correlation of SES and intention of smoking

Table 2 presents the correlations of the potential mediators, the control variables and the main outcome variables. Intention to smoke was positively correlated with advantages and negatively with disadvantages toward smoking (advantages: *r* = 0.21 (*p* < 0.01); disadvantages: *r* = -0.18 (*p* < 0.01)), a low subjective norm of people in the social environment perceived by the child (*r* = -0.20 (*p* < 0.01)), low self-efficacy expectations to refuse a cigarette (*r* = -0.15 (*p* < 0.01)) and more people who smoke in the environment of the child (*r* = 0.16 (*p* < 0.01)). In the HSES environment fewer people were smokers (*r* = -0.10 (*p* < 0.01)) and children living in a HSES environment had more often the intention not to smoke (*r* = -0.05 (*p* = 0.01)).

**Table 2:** Spearman correlations of potential mediators and confounders of intention to smoke

	**1.**	**2.**	**3.**	**4.**	**5.**	**6.**	**7.**	**8.**	**9.**	**10.**
**1. Attitude (advantages)**	1									
**2. Attitude (disadvantages)**	-0.27**	1								
**3. Subjective norm**	-0.25**	0.32**	1							
**4. Self-efficacy**	-0.34**	0.24**	-0.22**	1						
**5. Modelling**	0.16**	-0.13**	-0.14**	-0.09**	1					
**6. Age**	-0.01	0.02	0.04	-0.04*	0.09**	1				
**7. Ethnicity**	-0.04*	0.07**	0.03	0.02	0.06**	0.04*	1			
**8. Gender**	0.02	0.04*	0.06**	-0.01	-0.01	-0.05*	0.05**	1		
**9. SES**	-0.02	-0.02	0.01	<0.01	-0.10**	-0.03	-0.04	0.01	1	
**10. Intention**	0.21**	-0.18**	-0.20**	-0.15**	0.16**	0.02	-0.01	-0.06**	-0.05*	1

*Significant at 0.05 level (2-tailed).

**Significant at 0.01 level (2-tailed).

### Individual cognitive and social environmental mediators

**Figure 1:**
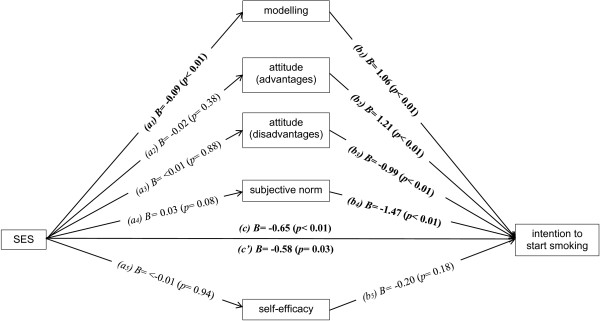
**Multiple mediation model assessing potential social environmental and individual cognitive mediators between SES and the intention to start smoking.** Note: *a* = association of independent factor and potential mediator; *b* = association of potential mediator and dependent factor; *c* = association between dependent and independent factor; *c’* = association between dependent and independent factor adjusted for potential mediators.

A multiple mediation model including the potential social environmental (modelling) and individual cognitive mediators (attitude (advantages); attitude (disadvantages); subjective norm; self-efficacy) is presented in Figure 1. Only for modelling both pathway *a*_*1*_ representing the association between SES and modelling (*B* = -0.09 (*p* < 0.01)) and pathway *b*_*1*_ representing the association between modelling and intention to smoke (*B* = 1.06 (*p* < 0.01)) were significant, indicating that modelling was the only variable that mediated the association between SES and intention to smoke [33]. The results show that there is partial mediation as indicated by the reduced, but still significant, pathway *c’* (*B* = -0.58 (*p* = 0.03)).

**Figure 2:**
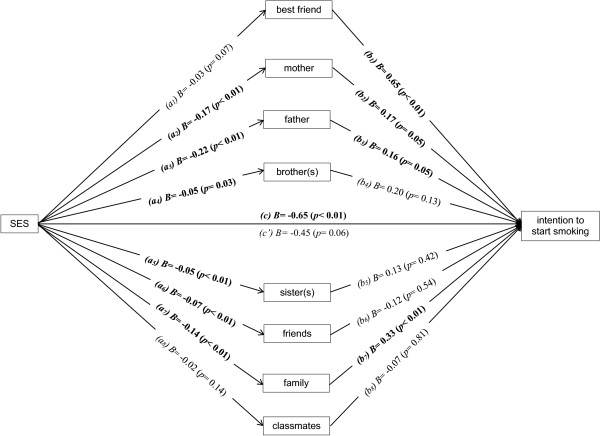
**Multiple mediation model assessing potential mediators of individual components of modelling between SES and the intention to start smoking.** Note: *a* = association of independent factor and potential mediator; *b* = association of potential mediator and dependent factor; *c* = association between dependent and independent factor; *c’* = association between dependent and independent factor adjusted for potential mediators.

Figure 2 presents a multiple mediation model including the individual components of modelling (best friend; mother; father; brother(s); sister(s); friends; family; classmates) to assess which item explained the mediation most. The smoking behaviour of the father (*B* = -0.22 (*p* < 0.01)); (*B* = 0.16 (*p* = 0.05)), mother (*B* = -0.17 (*p* < 0.01)); (*B* = 0.17 (*p* = 0.05)) and other family members (*B* = -0.14 (*p* < 0.01)); (*B* = 0.33 (*p* < 0.01)) mediated most of the association between the SES and intention to smoke. Partial mediation may be present since pathway *c’* indicates a borderline significant relationship (*B* = -0.45 (*p* = 0.06)).

Within the multiple mediation models no interaction effects (*p* > 0.10) were found for gender and the potential mediators indicating that gender was not a moderating factor between SES and the intention to smoke. For that reason the analyses stratified by gender were not further indicated.

## Discussion

The aim of the present study was to identify whether individual cognitions or social environmental factors can explain SES differences in the intention to start smoking, for Dutch primary school children (aged 10 – 11 years). The results indicated that modelling mediated the relation between SES and the intention to start smoking among primary school children in The Netherlands. Furthermore, cognitive aspects such as attitude, subjective norm and self-efficacy were not associated with the intention to engage in smoking. In-depth analyses showed that especially the smoking behaviour of the mother, father and other family members mediated the association between SES and the intention to engage in smoking.

In line with the results of previous studies we found that there was a difference in the intention to start smoking among children living in a HSES or LSES environment [10,23,35]. It has been suggested that this higher intention to start smoking (which is likely to result in actual smoking behaviour at an older age) is caused by the higher smoking prevalence of others in the social environment of children with a LSES [10,23,36]. Our results support this suggestion since we found indeed that the perceived smoking behaviour of others (i.e. modelling) was the only mediator for the difference in smoking intention between children of HSES and LSES environments. This finding is in line with the findings of former studies among adolescents where the smoking behaviour of parents and peers was found to be mediators between SES and adolescent smoking behaviour [23,24].

In this study we did not find support for cognitive variables (e.g. attitude, subjective norm and self-efficacy) being mediators of the association between SES and intention of smoking, which was demonstrated by the non-significant association between SES and these cognitions, however; the association between the individual cognitions and intention was significant. We studied these factors as potential mediators, since it has been demonstrated in a previous study that individual cognitions with respect to smoking or smoking initiation are less positive among LSES youngsters [10]. They had more positive attitudes, perceived more positive subjective norms and had lower self-efficacy expectations toward smoking, compared to HSES youngsters. However, this difference should be interpreted with caution since the present study used a cross-sectional design in which no firm conclusions can be made concerning causal relationships between SES, intention of smoking and the cognitive variables. The lack of support of cognitive variables being potential mediating variables may be explained by the younger age of children in the present study, as compared to prior studies [10,23]. Another explanation may be that SES was measured on a neighbourhood level, whereas others were able to measure SES on a parental level [10,11].

Parents who smoke are assumed to influence their children’s smoking behaviour and their intention to start smoking. It has been reported that children of smoking parents are at a higher risk of intending to start or to actually start smoking [22,37,38], especially in LSES families [39], but no conclusive evidence has been found on whether fathers or mothers are most influential on whether their children engage in smoking [39]. In the present study we also found that the smoking behaviour of both fathers and mothers were among the most important mediators between SES and intention to smoke.

Boys and girls may be differently influenced to engage in smoking [40]. A mother, for example, is reported in previous studies to influence their daughters’ behaviour, not only in smoking but also in their eating behaviour [41] or the onset of sexual behaviour [42]. On the other hand, boys’ behaviour is expected to be more influenced by the behaviour and opinions of their (best) friends [15,19]. The results of the present study showed that gender did not moderate the association between SES and intention to smoke and the potential mediators, indicating that there are no differences between boys and girls in mediating factors. These results are similar to the findings reported by Ausems et al. [43] in which no gender-specific predictors of later smoking initiation were found. Although more research is needed to investigate if a gender-specific approach or a family-directed approach will be beneficial to decrease smoking initiation among the young.

This exploratory study shows that especially factors in the social environment may partially explain the differences in intention to start smoking among children of LSES and HSES environments. Previous studies that have studied the behaviour of others as social environmental factors (modelling) have reported similar findings of the social environment being highly influential on whether children and adolescents engage in smoking [20,21,23]. Smoking among LSES children is likely to be higher compared to HSES children, since they live more often in LSES neighbourhoods in which smoking is more prevalent [10]. To gain more insight into the influence of the (social) environmental factors on the explanation of smoking intention and behaviour, future studies could better use an objective measure of the behaviour of others. Since smoking in the age group under study (10 – 11 years) is fairly low [1] we used the intention to engage in smoking as the main outcome measure. This may be a weakness, since we do not know whether intention to smoke at this age will translate to actual smoking at a later age, but evidence suggests that there is a fairly strong association between intention and behaviour at a later age [44,45].

### Strengths and limitations

A strength of the present study was the large and diverse sample size. A total of 162 primary schools participated across all regions in The Netherlands. But this study was also subject to several limitations. Since mediation analyses were conducted on the cross-sectional data of the ‘Fun without Smokes’ study several drawbacks may be present such as reverse causation or the inability to interpret results as causal relationships. However, due to the use of SES and intention to start smoking it is conceptually not possible that there is reverse causation in our data. Nevertheless, no firm conclusion can be made based on our findings but certainly some implications for future research can be given. About 3,500 Dutch primary schools were approached to participate in the present study, but only 162 schools were able to participate (4.6%). For that reason data from this study originates from a selective sample and the results can only be applied to the children of the participating schools. Furthermore, SES was measured in this exploratory study on a neighbourhood level (based on children’s 4-digit postal code). Even though this is not similar to an individual indicator of SES, it is highly likely that the individual SES of the children is the same as the neighbourhood SES, since the neighbourhood SES is based on the SES of the individuals living in that neighbourhood. Nevertheless, it is advisable for future research to repeat the procedure of the present study with an individual SES measure for the participating children.

## Conclusions

In this study we found that modelling could explain the association between SES and intention to start smoking. The smoking behaviour of the father, mother and other family members was shown to be most influential concerning the intention to engage in smoking for children living in a LSES environment. It may be important that future smoking prevention programmes focus on environmental factors (i.e. the smoking behaviour of parents and family members) to decrease smoking onset in children of LSES environments, but support with more evidence is also needed.

## Competing interests

Hein de Vries is scientific director of Vision2Health, a company that licenses evidence-based innovative computer-tailored health communication tools. The other authors declare that they have no competing interests.

## Authors’ contributions

HPC significantly contributed to writing this paper, while LM, AO and HdV were involved in revising the manuscript critically. MC advised on the statistical analyses and was involved in revising the manuscript. All authors read and approved the final version of the manuscript.

## Pre-publication history

The pre-publication history for this paper can be accessed here:

http://www.biomedcentral.com/1471-2458/14/191/prepub
